# Supplementary figures and images for: Hypothalamic transcriptome analysis reveals the neuroendocrine mechanisms in controlling broodiness of Muscovy duck (Cairina moschata)
Source: PLoS One. 2019 May 9;14(5):e0207050. doi: 10.1371/journal.pone.0207050 (PMC6508920; doi:10.1371/journal.pone.0207050)

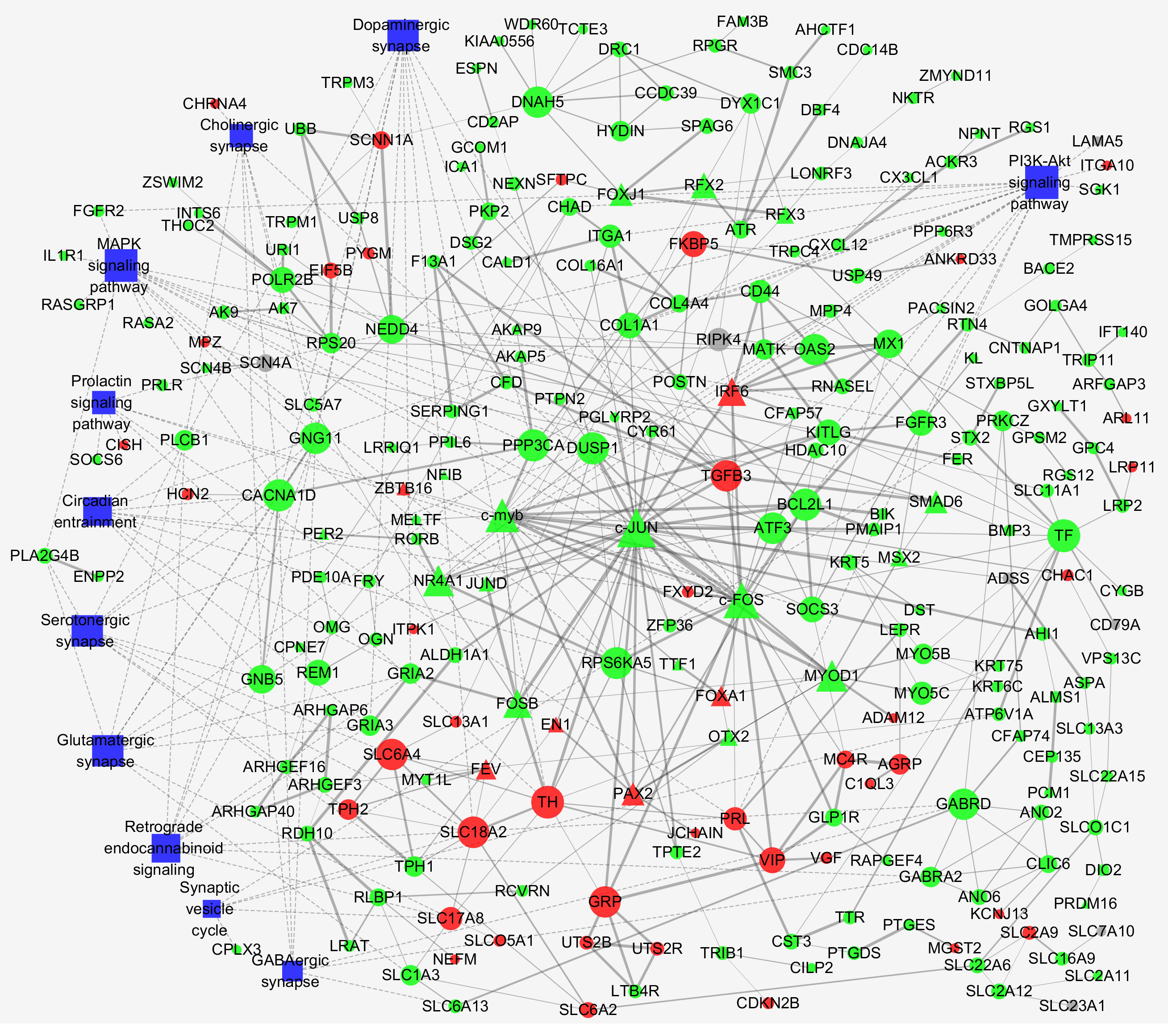

Supplement: S1 Fig — (TIF) [file pone.0207050.s001.tif]
